# Intrauterine ozone therapy reduces bacterial load in mares with mixed bacterial endometritis: a longitudinal study

**DOI:** 10.1007/s11259-026-11438-3

**Published:** 2026-07-31

**Authors:** Diego Guedes Campos, Reno Roldi Araujo, Suzana Rossato Feltrin, Jorge Henrique Villela Botelho, Juliana Felipetto Cargnelutti, Valério Marques Portela, Carolina dos Santos Amaral, Alfredo Quites Antoniazzi

**Affiliations:** 1 Laboratory of Biotechnology and Animal Reproduction (BioRep), Federal University of Santa Maria (UFSM), Santa Maria, 97105-900 RS Brazil; 2 GeneTech Animal Reproduction, Campinas, 13080-650 SP Brazil; 3 Universidade Professor Edson Antonio Velano, Alfenas, Minas Gerais Brazil; 4 Laboratory of Bacteriology (LABAC), Department of Microbiology and Parasitology, Federal University of Santa Maria (UFSM), Santa Maria, 97105-900 RS Brazil

**Keywords:** Colony-forming units, Polymicrobial infection, Neutrophil infiltration, Non-antibiotic therapy, Uterine microbiology

## Abstract

Mixed bacterial endometritis poses a therapeutic challenge in mares due to the coexistence of multiple pathogens with divergent antimicrobial susceptibility profiles. This study evaluated the effects of intrauterine ozone insufflation on uterine bacterial load and endometrial inflammatory response in mares with naturally acquired mixed bacterial endometritis. Fifteen mares with mixed bacterial infection were allocated to two treatment groups: a pathological control group receiving pure medical oxygen insufflation (PC; *n* = 6) and an ozone-treated group receiving 42 µg/mL ozone for three consecutive days (O3; *n* = 9). Eight clinically healthy mares subjected to the same ozone protocol served as a safety control group. Bacterial load was quantified in colony-forming units per milliliter (CFU/mL) from low-volume uterine flushings obtained at baseline (C1D0), six days post-treatment (C1D6), and at the onset of the subsequent estrous cycle (C2D0). A linear mixed model revealed significant group-by-time interactions for both bacterial load and neutrophil counts (*P* < 0.001). At C1D6, the O3 group showed a median 100% reduction in bacterial load (from 65,000 to 0 CFU/mL; *P* < 0.001), a culture positivity rate of 22.2% not significantly different from healthy controls (*P* = 0.260) and an 88.9% reduction in neutrophil counts. At C2D0, although bacterial cultures became positive again in all mares in the O3 group, bacterial load remained 77.5% below baseline (*P* < 0.001), and neutrophil counts remained 42.4% below baseline, indicating a sustained biological response following treatment. The PC group showed no microbiological or inflammatory variation throughout follow-up. The healthy control group remained culture-negative at all time points. Intrauterine ozone insufflation substantially reduced bacterial load and endometrial neutrophil infiltration in mares with mixed bacterial endometritis, without inducing bacterial colonization in healthy animals.

**Supplementary Information:**

The online version contains supplementary material available at https://doi.org/10.1007/s11259-026-11438-3.

## Introduction

Infectious endometritis remains one of the leading causes of subfertility in mares, impairing fertility, embryo survival, and reproductive efficiency in equine breeding populations (LeBlanc and Causey 2009; Troedsson and Woodward 2016). The condition arises from the failure of the uterus to adequately clear microorganisms and inflammatory products following natural mating or artificial insemination, promoting persistent endometrial inflammation and alterations of the uterine environment consistent with recurrent reproductive failure (LeBlanc 2010; Christoffersen and Troedsson 2017).

Several aerobic microorganisms have been associated with equine endometritis, including *Streptococcus equi* subsp. *zooepidemicus*, *Escherichia coli*, *Klebsiella pneumoniae*, *Pseudomonas aeruginosa*, and various *Staphylococcus* species (LeBlanc 2010; Ferrer and Palomares 2018; Díaz-Bertrana et al. 2021). Although many studies describe infections caused by a single bacterial agent, evidence indicates that mixed bacterial infections also occur frequently in equine uterine disease, although their reported prevalence varies considerably across populations and clinical settings. In a retrospective series of 8,296 uterine samples from breeding mares in Florida, USA, Davis et al. (2013) identified growth of two or more pathogens in 12.6% of positive cultures, with *E. coli* being the organism most frequently associated with co-infections, predominantly in combination with *S. equi* subsp. *zooepidemicus*, *K. pneumoniae*, and *P. aeruginosa*.

Ferrer and Palomares (2018) reported mixed bacterial growth in 62.2% of mares with post-partum metritis, whereas Díaz-Bertrana et al. (2021) observed mixed growth in approximately 7% of positive uterine samples collected under field conditions. Supporting these findings, Nunes et al. (2026) identified *Corynebacterium uterequi* in uterine samples from mares with reproductive disorders in Rio Grande do Sul, Brazil; in half of the cases, the organism was isolated in co-infection with other bacterial agents, including *Streptococcus* sp., *Arcanobacterium* sp., and *Morganella* sp., with moderate-to-severe endometrial inflammation observed more frequently in mares with mixed bacterial infection than in those with single-agent isolation.

The simultaneous presence of multiple microorganisms in the uterine environment imposes therapeutic challenges beyond those associated with single-agent infections. Complex microbial interactions may promote bacterial persistence, mutual protection against host defense mechanisms, and divergent antimicrobial susceptibility profiles, making therapeutic outcomes less predictable (Ferrer and Palomares 2018). Furthermore, Gram-negative bacteria frequently associated with mixed bacterial infections, such as *E. coli* and *Klebsiella* spp., are of growing clinical concern due to the high frequency of antimicrobial resistance observed among equine uterine isolates (Ferrer and Palomares 2018; Díaz-Bertrana et al. 2021).

Conventional treatment of bacterial endometritis relies primarily on the combination of uterine lavage, ecbolic administration, and systemic or intrauterine antimicrobial therapy (LeBlanc 2010; Christoffersen and Troedsson 2017). However, the repeated use of antimicrobials has been questioned considering the global rise in bacterial resistance and the need for therapeutic strategies that reduce the selective pressure exerted by these drugs (Díaz-Bertrana et al. 2021). In this context, complementary or alternative therapies capable of reducing microbial load without direct antibiotic use have emerged as a clinically relevant approach in equine reproductive medicine.

Among these alternatives, intrauterine ozone therapy has received attention for its antimicrobial, immunomodulatory, and potentially anti-inflammatory properties (Bocci 2011; Elvis and Ekta 2011). Ozone exerts its effects through oxidative mechanisms capable of damaging microbial cell membranes, proteins, and nucleic acids, thereby reducing the viability of a broad range of infectious agents (Bocci 2011). Experimental studies have demonstrated antimicrobial activity of ozone against pathogens associated with equine endometritis, including isolates resistant to conventional antimicrobials (Ávila et al. 2022; Köhne et al. 2023).

Recent studies have shown that intrauterine ozone administration can reduce endometrial inflammation and microbiological positivity without inducing relevant histological changes in the equine endometrium (Donato et al. 2023, 2024). However, most available investigations evaluated heterogeneous populations without distinguishing between single-agent and mixed bacterial infections, and no study has longitudinally quantified uterine bacterial load in CFU/mL as a primary outcome. It therefore remains unknown whether the microbiological and anti-inflammatory effects attributed to ozone therapy are similarly observed in mares with mixed bacterial endometritis, a condition recognized as therapeutically more complex and challenging.

The present study evaluated the effects of intrauterine ozone insufflation on uterine bacterial load, quantified longitudinally in CFU/mL across consecutive estrous cycles, and on the endometrial inflammatory response in mares with naturally acquired mixed bacterial endometritis. Qualitative changes in uterine bacterial composition were investigated as a secondary outcome.

## Materials and methods

### Ethical approval and study site

The study was conducted between November 2025 and March 2026 (approximately five months), during the 2025–2026 breeding season, at a commercial Mangalarga Marchador embryo transfer facility located in southern Minas Gerais state, Brazil (21°50′38″ S, 44°58′35″ W). All procedures involving animals were approved by the Animal Ethics Committee of the Universidade José do Rosário Vellano (CEUA/UNIFENAS; protocol no. 05 A/2022) and were conducted in accordance with institutional and national guidelines for animal care and use.

### Animals and eligibility criteria

A total of 57 Mangalarga Marchador embryo recipient mares enrolled in the commercial embryo transfer program at the same commercial facility where the study was conducted were evaluated for study eligibility during the breeding season. All mares had a documented history of subfertility, characterized by repeated failure to establish pregnancy over at least two consecutive breeding seasons, with or without persistent intrauterine fluid accumulation following natural mating or artificial insemination. Selection criteria were based on regular estrous cyclicity and documented reproductive history, and body condition score (BCS) was assessed according to Carroll and Huntington (1988). Only mares with a BCS between 4 and 7 on a 1-to-9 scale were included. Mares were excluded if they presented systemic disease, severe structural abnormalities of the reproductive tract identified on transrectal palpation or ultrasonography, BCS outside the established range, or had received antimicrobial treatment within 30 days prior to the onset of the experimental protocol.

Throughout the experimental period, all mares were maintained under a semi-intensive management system with free access to pasture, water, and mineral supplementation under identical nutritional and reproductive management conditions.

### Reproductive screening and endometritis diagnosis

Approximately 30 days before the onset of the experimental protocol, all 57 mares underwent a complete reproductive evaluation, including perineal conformation inspection, cervical assessment by vaginoscopy, transrectal palpation, and ultrasonographic examination of the reproductive tract. Intrauterine fluid detected on ultrasonography was recorded as a complementary clinical variable but was not adopted as a diagnostic criterion for endometritis or as a primary therapeutic outcome. Endometritis was diagnosed exclusively when positive inflammatory cytology and growth of a potentially pathogenic bacterium were detected simultaneously in the same sample Uterine collections were not scheduled on a predetermined day of the estrous cycle but were performed only after biological confirmation of estrus, established by transrectal ultrasonography based on the presence of characteristic endometrial edema, a dominant follicle (≥ 28 mm), absence of a corpus luteum, and cervical relaxation.

Mares were restrained in stocks and the tail was wrapped. The perineal region was cleansed with neutral soap, followed by antisepsis with chlorhexidine and 10% povidone-iodine solution, triple rinsing with sterile water, and drying with sterile disposable material. The uterine catheter was protected within the sterile inner surface of an obstetric glove throughout manual passage through the vestibule, vagina, and cervical canal, minimizing contact with the vaginal and cervical flora prior to entry into the uterine lumen (LeBlanc et al. 2007; Linton and Sertich 2016).

Uterine samples were obtained by low-volume uterine lavage for simultaneous cytological and microbiological evaluation. Cytological smears were prepared from the recovered fluid, stained using the Rapid Panoptic method, and evaluated under light microscopy at 400× magnification by a single trained evaluator blinded to microbiological results. Ten consecutive non-overlapping high-power fields were assessed per slide, avoiding areas with excessive cellular material or staining artifacts, and results were expressed as the mean number of polymorphonuclear neutrophils (PMNs) per field. Samples containing more than two PMNs per high-power field were considered indicative of uterine inflammation, in accordance with previously established criteria for mares (Cocchia et al. 2012; LeBlanc 2010).

Applying these diagnostic criteria during the screening evaluation, 17 of the 57 mares were diagnosed with mixed bacterial endometritis, defined as the simultaneous isolation of two or more bacterial species from the same uterine sample, whereas the remaining 40 mares presented single-agent infections. Only mares diagnosed with mixed bacterial endometritis at screening were considered eligible for inclusion in the study and subsequent analyses. Eligibility was confirmed by repeating the cytological and microbiological evaluations at the onset of the experimental protocol.

### Experimental design and group allocation

The experiment was conducted over two consecutive estrous cycles (Cycle 1 and Cycle 2; C1 and C2), allowing longitudinal monitoring of microbiological and inflammatory dynamics following treatment. Sample size was determined by the availability of eligible animals identified at screening, given that naturally acquired mixed bacterial infections represent a lower-prevalence subpopulation relative to single-agent infections, as reported in the literature (Díaz-Bertrana et al. 2021; Davis et al. 2013).

Of the 17 mares diagnosed with mixed bacterial infection, 15 completed all protocol evaluations and were included in the final analysis. Two mares initially allocated to the pathological control group were withdrawn: one developed acute hindlimb lameness during the experimental period requiring pharmacological intervention incompatible with protocol continuation, and the other failed to return to estrus within the expected experimental window following dinoprost tromethamine administration during the intercycle period, precluding sample collection at C2D0.

The 15 mares with mixed bacterial infection were randomly allocated by simple drawing to two experimental groups: a pathological control group receiving intrauterine pure medical oxygen insufflation (PC; *n* = 6; mean age: 11.5 ± 1.9 years) and an ozone-treated group receiving intrauterine ozone insufflation (O3; *n* = 9; mean age: 10.6 ± 2.5 years). Allocation was stratified by farm of origin to control for potential locality effects and was performed independently of the bacterial species identified in uterine culture, baseline microbial load, or cytological findings.

Additionally, a group of clinically healthy recipient mares from the same commercial herd was included as a biological safety control (Control; *n* = 8; mean age: 7.4 ± 1.3 years). These mares showed no clinical, ultrasonographic, cytological, or microbiological evidence of endometritis at screening and received intrauterine ozone insufflation under the same parameters applied to the O3 group, as described in Sect.  2.5. Participation of these mares was scheduled during intervals between embryo transfer cycles, which conditioned the number of available animals and the duration of protocol participation.

This group was included to determine whether intrauterine ozone administration could induce adverse changes in a healthy endometrium and was not used as a primary comparative group for therapeutic efficacy assessment in mares with endometritis. Accordingly, all analyses related to treatment response were conducted exclusively between groups comprising mares with mixed bacterial infections (PC and O3).

The inclusion of clinically healthy mares for biological safety assessment of intrauterine interventions follows an approach similar to that employed by Beckers et al. (2025), who investigated the effects of intrauterine ceftiofur infusion on the uterine microbiome of healthy mares to determine potential treatment-induced alterations in a uterine environment without evidence of disease. The Control group was therefore included exclusively for microbiological and inflammatory safety monitoring of intrauterine ozone therapy and not for therapeutic efficacy comparisons.

### Treatment protocol

Treatment was administered over three consecutive days (D1 to D3), beginning the day after baseline sample collection (C1D0). Mares in the O3 and Control groups received intrauterine ozone insufflation at a concentration of 42 µg/mL, delivered at a flow rate of 0.25 L/min for five minutes, totaling approximately 1.25 L of gas per application. Mares in the PC group received intrauterine pure medical oxygen insufflation under identical operational parameters, ensuring procedural equivalence between groups and blinding of the evaluator responsible for subsequent microbiological and cytological assessments.

Ozone was generated from medical-grade oxygen (99.5%) using a commercial generator (Ozone & Life^®^, model O&L 3.0 RM, Brazil), with continuous concentration monitoring by ultraviolet photometry integrated into the device. The ozone concentration and exposure time were adapted from protocols previously described for the treatment of equine endometritis, based on studies demonstrating antimicrobial activity and endometrial safety (Ávila et al. 2022; Köhne et al. 2023; Donato et al. 2024; Botelho et al. 2024).

### Evaluation points and sample collection schedule

Evaluations were performed at three predefined experimental time points: C1D0 (baseline evaluation, performed immediately before the first ozone application after biological confirmation of estrus, based on the presence of characteristic endometrial edema, a dominant follicle of approximately 28 mm in diameter, cervical relaxation, and absence of a corpus luteum), C1D6 (the second evaluation, performed six days after C1D0 and three days after the final ozone application), and C2D0 (the evaluation performed at the onset of the subsequent estrous cycle). C1D6 was established as the predefined post-treatment evaluation to assess the short-term response to ozone therapy. Based on the experimental timeline, mares were expected to be in the periovulatory period, corresponding to the transition from late estrus to early diestrus. Because the timing of ovulation varies naturally among mares, slight individual variation in reproductive stage at C1D6 was anticipated and considered part of the experimental design. After C1D6 sample collection, mares underwent transrectal ultrasonographic monitoring until ovulation was confirmed individually. Thereafter, ultrasonographic examinations were performed at 48-hour intervals to monitor uterine and ovarian status throughout diestrus. Day 8 after ovulation was adopted as the standardized time point for induction of luteolysis in all mares, ensuring a consistent interval before synchronization of the subsequent estrous cycle. Immediately before dinoprost tromethamine administration (5 mg, IM; Lutalyse^®^, Zoetis, Brazil), each mare underwent transrectal ultrasonography to confirm the presence of a functional corpus luteum and persistence of diestrus. Although mares with endometritis may exhibit variable or shortened diestrous periods, this individualized ultrasonographic monitoring ensured that prostaglandin was administered only after confirmation of the appropriate reproductive stage. Following dinoprost administration, transrectal ultrasonographic examinations continued at 48-hour intervals until biological confirmation of estrus. As in C1D0, C2D0 sampling was performed only after confirmation of the same biological criteria for estrus (characteristic endometrial edema, a dominant follicle of approximately 28 mm in diameter, cervical relaxation, and absence of a corpus luteum). No treatment was administered at C2D0; only uterine samples were collected for microbiological and cytological evaluation at the beginning of the subsequent estrous cycle. Consequently, both C1D0 and C2D0 were standardized according to the biological reproductive status of each mare rather than to a fixed interval after prostaglandin administration.

### Uterine sample collection and processing

Uterine sample collection was performed by low-volume uterine lavage at all experimental time points (C1D0, C1D6, and C2D0), as previously described (LeBlanc et al. 2007; Linton and Sertich 2016), with adaptations regarding the infused volume and the use of oxytocin to optimize effluent recovery. A total of 200 mL of sterile lactated Ringer’s solution was infused into the uterine lumen through a transcervically positioned uterine catheter, followed by transrectal uterine massage to promote uniform fluid distribution throughout the uterine body and horns, as similarly described by Heil et al. (2023), with adaptation of the infused volume. Two minutes after infusion, oxytocin (20 IU, IV) was administered to facilitate myometrial contraction and uterine content recovery, as described by Linton and Sertich (2016). This procedure was standardized across all collections and groups and was not considered part of the therapeutic protocol.

Approximately 100 mL of uterine effluent was recovered per mare at each experimental time point. An aliquot was immediately used for cytological smear preparation. The remaining volume was centrifuged at 300 × g for 10 min at 4 °C, and the resulting pellet was resuspended in sterile physiological saline to a standardized final volume of 50 mL in a sterile Falcon tube. Samples were maintained at 4 °C and transported to a single laboratory for microbiological processing within a maximum of eight hours after collection, ensuring standardization and comparability across all experimental time points.

### Internal contamination control

To verify the microbiological integrity of the collection system, control samples of lactated Ringer’s solution were obtained at each experimental time point (C1D0, C1D6, and C2D0) in all groups, immediately before introduction of the uterine catheter into the uterine lumen. Fluid was collected directly from the distal tip of the catheter following complete purging of the infusion system (tubing and connections) into a sterile container, without contact with any anatomical structures of the mare. These control samples were subjected to the same microbiological procedures applied to uterine samples including centrifugation, resuspension, and inoculation onto culture media and were processed simultaneously under identical laboratory conditions by the same operator.

The absence of bacterial growth in all control samples confirmed the sterility of the infusion system throughout the experimental period, excluding iatrogenic contamination originating from the collection system as a source of the microorganisms identified in uterine samples and ensuring that microbiological results reflect the intrinsic bacterial content of the uterine environment evaluated.

### Bacterial culture and microbiological analysis

Uterine samples were subjected to aerobic bacterial culture for quantitative and qualitative characterization of microorganisms present in the uterine environment. To enhance diagnostic sensitivity and favor recovery of multiple bacterial species potentially present in the same sample, samples were homogenized and centrifuged prior to microbiological plating, with the resulting pellet resuspended in sterile physiological saline before inoculation.

Bacterial quantification was performed by decimal serial dilutions (10⁻¹ to 10⁻⁴) of the resuspended pellet, with 100 µL aliquots inoculated onto Plate Count Agar (PCA) and incubated at 37 °C for 24 h, with continued observation for up to 72 h in the absence of initial growth. Colony counts were performed on plates containing between 30 and 300 colony-forming units, and results were expressed in CFU/mL after correction for inoculated volume, dilution factor, and sample concentration factor. Samples with no detectable growth after the incubation period were classified as culture-negative and assigned a value of 0 CFU/mL for quantitative analyses. For samples with mixed bacterial growth, CFU/mL quantification was based on the total bacterial count observed on PCA plates, considering all recovered colony morphotypes collectively. The individual contribution of each bacterial species was not used as a primary quantitative outcome and was recorded solely for qualitative analyses of microbiological composition and longitudinal isolate behavior.

Additional aliquots were inoculated onto Brain Heart Infusion (BHI) agar supplemented with 5% equine blood to favor recovery of potentially fastidious microorganisms, with incubation under aerobic conditions for up to 72 h. Bacterial growth was classified into three categories based on results from both culture media combined: absence of growth, single-agent isolation, or mixed flora. For diagnostic purposes, growth was considered positive when microorganisms recognized as potentially pathogenic to the equine reproductive tract were identified, including beta-hemolytic organisms, enteric Gram-negative bacilli, and other clinically relevant isolates, in accordance with criteria described in the literature (LeBlanc and Causey 2009; Díaz-Bertrana et al. 2021). Microorganism identification was performed by MALDI-TOF mass spectrometry and, when necessary, complemented by Gram staining and conventional biochemical testing according to established laboratory protocols.

For the purposes of the present study, mixed bacterial infection was defined as the simultaneous isolation of two or more bacterial species from the same uterine sample, regardless of the culture medium from which they were recovered. Longitudinal analyses focused on the assessment of persistence, elimination, replacement, or emergence of bacterial species across experimental time points, as well as changes in the number of species isolated per mare between consecutive estrous cycles. Microbiological recurrence was defined as the reappearance of positive bacterial growth at C2D0 in mares that had presented negative cultures at C1D6, regardless of the identity of the isolated species.

Antimicrobial susceptibility testing was performed by the disk diffusion method (Kirby-Bauer) in accordance with Clinical and Laboratory Standards Institute recommendations (CLSI 2021), with results interpreted as susceptible, intermediate, or resistant. This test was conducted exclusively for complementary characterization of bacterial isolates and did not constitute a primary study outcome. Anaerobic and fungal cultures were not performed, as the primary objective was the longitudinal quantitative assessment of aerobic bacterial load associated with mixed bacterial infections in mares with endometritis under field conditions.

### Statistical Analysis

Statistical analyses were performed using IBM SPSS Statistics version 26 (IBM Corp., Armonk, NY, USA). Continuous variables were expressed as mean ± SD or median (IQR; Q1–Q3) according to data distribution, and categorical variables as absolute and relative frequencies.

Normality was assessed using the Shapiro–Wilk test complemented by histogram inspection. Bacterial load (CFU/mL) and neutrophil count showed pronounced asymmetry and were log-transformed using log₁₀(X + 1) prior to inferential analyses. Age differences among groups were evaluated by one-way ANOVA with Tukey’s post-hoc test. Culture positivity was compared among groups using Fisher’s exact test and within groups over time using the corrected McNemar’s test.

Longitudinal changes in bacterial load and neutrophil count were analyzed by linear mixed-effects models for repeated measures fitted by REML, with treatment group, experimental time point, and their interaction as fixed effects, and mare as a random intercept. Pairwise comparisons were derived from predefined contrasts specified prior to data analysis, controlling the number of inferential tests performed and reducing the risk of Type I error inflation. Statistical significance was set at *P* < 0.05.

The healthy control group was included exclusively for biological safety assessment and was not included in primary efficacy analyses, which were conducted solely between the PC and O3 groups.

## Results

None of the mares showed adverse reactions to ozone administration throughout the experimental period. The sterility of the collection system was confirmed by the absence of bacterial growth in all internal controls across all assessed timepoints, ruling out iatrogenic contamination as a source of bacterial growth in the uterine samples.

### Microbiological findings

**Fig. 1:**
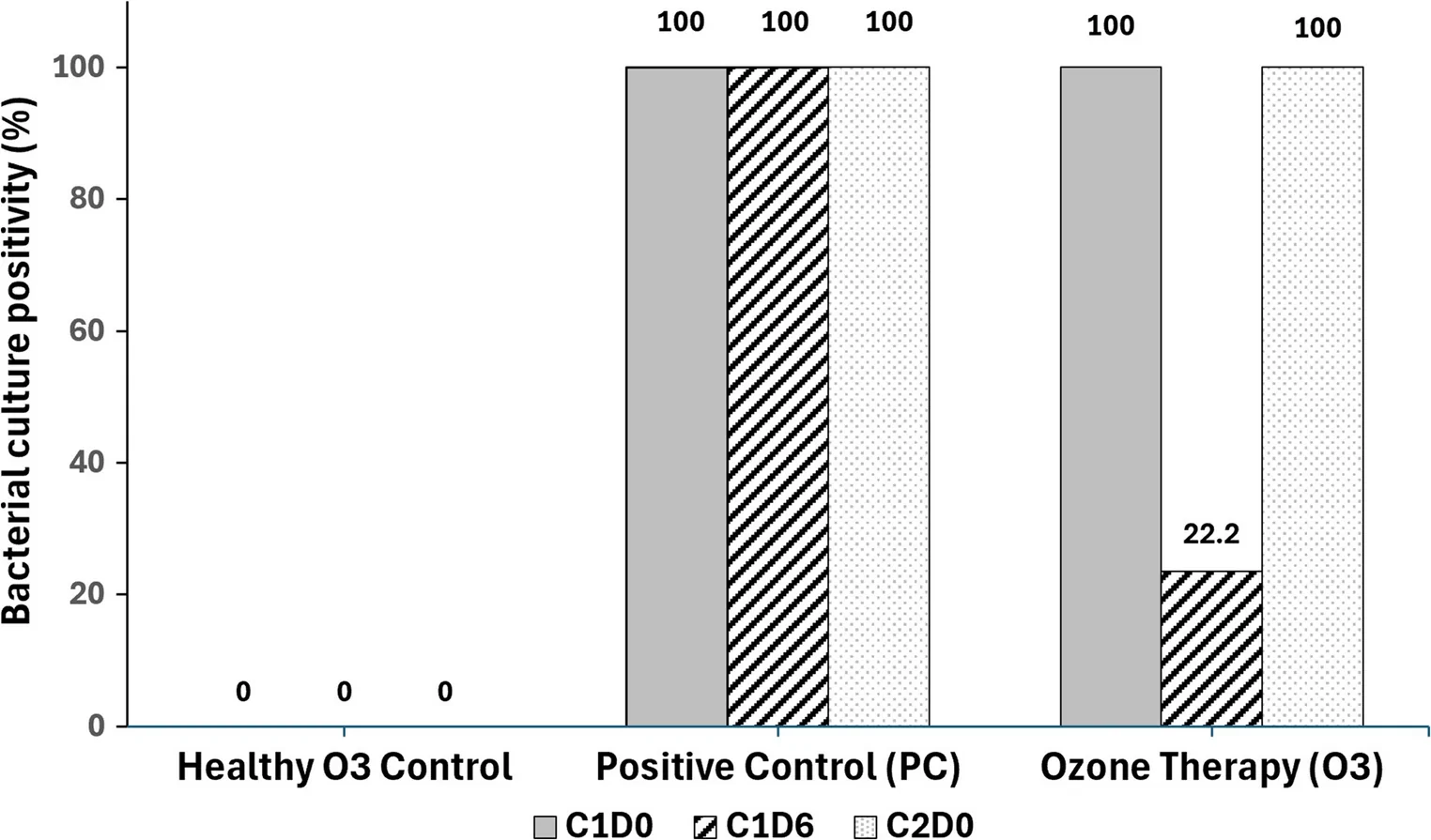
Uterine bacterial culture positivity (%) in clinically healthy mares submitted to intrauterine ozone insufflation (Healthy Control; *n* = 8), mares with mixed bacterial endometritis treated with pure medical oxygen (PC; *n* = 6), and mares with mixed bacterial endometritis treated with intrauterine ozone therapy (O3; *n* = 9), at baseline (C1D0), six days after treatment (C1D6), and at the beginning of the subsequent estrous cycle (C2D0). Comparisons between groups at each timepoint were performed using Fisher’s exact test, and within-group comparisons over time using McNemar’s exact test (*p* < 0.05)

The Control group remained free of bacterial growth at all assessment timepoints (C1D0, C1D6, and C2D0), demonstrating that intrauterine ozone insufflation did not induce bacterial colonization in clinically healthy mares (Fig. 1).

At baseline (C1D0), uterine cultures were 100% positive in both the PC and O3 groups (*p* = 1.000), confirming the microbiological equivalence between them before treatment. Following ozone therapy, a marked reduction in culture positivity was observed in the O3 group at C1D6 (22.2%), whereas the PC group remained 100% positive (*p* = 0.005). At the same point, the O3 group did not differ from the Control group (*p* = 0.260), whereas the PC group remained significantly different from the Control group (*p* = 0.0003). At C2D0, both groups were again 100% positive, with no difference between them (*p* = 1.000), although both remained significantly different from the Control group (PC: *p* = 0.0003; O3: *p* < 0.0001).

Within-group analysis showed significant temporal changes in the O3 group between C1D0 and C1D6 (*p* = 0.015) and between C1D6 and C2D0 (*p* = 0.015), whereas no significant changes were observed in the PC group, indicating persistent bacterial colonization.

The distribution of bacterial species identified in the O3 and PC groups across the three assessment timepoints is presented in Table 1. In the O3 group, *Escherichia coli* (27.8%) and *Klebsiella pneumoniae* (22.2%) were the predominant microorganisms at C1D0, a pattern that was repeated at C2D0 with similar frequencies (27.8% and 22.2%, respectively). *Staphylococcus* spp. and *Streptococcus equi* subsp. *zooepidemicus* accounted for 16.7% of occurrences at C1D0, decreasing to 11.1% at C2D0. *Pseudomonas aeruginosa* was identified at low frequency at C1D0 and C2D0 (5.6% each), while *Serratia* spp. was detected exclusively at C1D0 (5.6%), not being recovered at subsequent timepoints. At C1D6, bacterial diversity decreased markedly, with 88.9% of samples showing no detectable microbial growth; the only isolates identified at this timepoint were *E. coli* and *Staphylococcus* spp., each accounting for 5.6% of occurrences.

In the PC group, the microbiological profile remained stable with no negative cultures across all assessed timepoints. *Escherichia coli* was the most frequent microorganism (33.3%), followed by *Klebsiella pneumoniae* (25.0%), *Staphylococcus* spp. and *Streptococcus equi* subsp. *zooepidemicus* (16.7% each), and *Streptococcus* spp. (8.3%). *Pseudomonas aeruginosa* and *Serratia* spp. were not isolated from this group at any assessment timepoint.

**Table 1:** Longitudinal distribution of bacterial species isolated from uterine samples of mares with mixed bacterial endometritis treated with intrauterine ozone therapy (O3) or pure medical oxygen (PC)

Bacterial culture	O3 Group						PC Group	
	C1D0		C1D6		C2D0		C1D0 = C1D6 = C2D0	
	n	%	n	%	n	%	n	%
Absent	0	0.0	16	88.9	3	16.7	0	0.0
*Escherichia coli*	5	27.8	1	5.6	5	27.8	4	33.3
*Klebsiella pneumoniae*	4	22.2	0	0.0	4	22.2	3	25.0
*Staphylococcus spp.*	3	16.7	1	5.6	2	11.1	2	16.7
*Streptococcus equi subsp. zooepidemicus*	3	16.7	0	0.0	2	11.1	2	16.7
*Streptococcus spp.*	1	5.6	0	0.0	1	5.6	1	8.3
*Pseudomonas aeruginosa*	1	5.6	0	0.0	1	5.6	0	0.0
*Serratia spp.*	1	5.6	0	0.0	0	0.0	0	0.0

Absolute (n) and relative (%) frequencies of bacterial species isolated from uterine samples of mares with mixed bacterial endometritis treated with intrauterine ozone therapy (O3; *n* = 9) or pure medical oxygen (PC; *n* = 6) at baseline (C1D0), six days post-treatment (C1D6), and at the onset of the subsequent estrous cycle (C2D0). Frequencies were calculated based on the total number of bacterial occurrences identified, considering up to two isolates per animal; the O3 group comprised a maximum of 18 occurrences and the PC group 12 occurrences. The PC group showed no microbiological variation across time points

### Bacterial load and neutrophil count – longitudinal analysis

Mixed linear model analysis revealed a significant interaction between group and time for both bacterial load (CFU/mL) and neutrophil count, indicating that the longitudinal trajectories of these variables differed between the PC and O3 groups throughout the experimental period. The complete results of the contrasts estimated by the model are presented in Table 2.

**Table 2:** Longitudinal analysis of bacterial load (CFU/mL) and neutrophil count in mares with mixed bacterial endometritis treated with intrauterine ozone therapy (O3) or pure medical oxygen (PC) using a linear mixed-effects model

Contrast / Interaction	CFU/mL		Neutrophil count	
	Estimate	*P* value	Estimate	*P* value
Group × time interaction				
O3 × C1D6	-4.24	< 0.001	-0.73	< 0.001
O3 × C2D0	-1.56	< 0.001	-0.36	< 0.001
Within-group contrasts — PC				
C1D0 vs. C1D6	-0.03	0.74	0.02	0.72
C1D0 vs. C2D0	-0.04	0.61	0.02	0.69
C1D6 vs. C2D0	-0.01	0.88	0	0.95
Within-group contrasts — O3				
C1D0 vs. C1D6	-4.24	< 0.001	-0.71	< 0.001
C1D0 vs. C2D0	-1.56	< 0.001	-0.34	< 0.001
C1D6 vs. C2D0	2.68	< 0.001	0.37	< 0.001
Between-group comparisons				
PC vs. O3 at C1D0	-0.22	0.68	-0.01	0.87
PC vs. O3 at C1D6	4.03	< 0.001	0.75	< 0.001
PC vs. O3 at C2D0	1.35	0.012	0.38	< 0.001

Estimates and *P* values for group × time interaction effects, within-group contrasts, and between-group comparisons derived from the linear mixed-effects model for bacterial load (CFU/mL) and neutrophil count in mares with mixed bacterial endometritis treated with intrauterine ozone therapy (O3; *n* = 9) or pure medical oxygen (PC; *n* = 6). The model included treatment group, experimental time point, and the group × time interaction as fixed effects, with mare fitted as a random intercept. Both variables were log₁₀(X + 1) transformed prior to modeling. O3 × C1D6 interaction: magnitude of the additional change observed in the O3 group at C1D6 relative to the PC group. O3 × C2D0 interaction: magnitude of the additional change observed in the O3 group at C2D0 relative to the PC group

Baseline bacterial load was comparable between groups at C1D0 (PC: median 51,550 CFU/mL, IQR 6,475–193,250; O3: median 65,000 CFU/mL, IQR 33,050–191,000; *P* = 0.68). The linear mixed model identified a significant group-by-time interaction for bacterial load, with significant interaction components at C1D6 (estimate = − 4.24; *P* < 0.001) and C2D0 (estimate = − 1.56; *P* < 0.001), indicating that bacterial load trajectories differed significantly between groups across the experimental period.

In the PC group, bacterial load remained stable throughout follow-up, with no significant differences among time points (C1D0 vs. C1D6: *P* = 0.74; C1D0 vs. C2D0: *P* = 0.61; C1D6 vs. C2D0: *P* = 0.88) and medians of approximately 51,550, 54,750, and 51,250 CFU/mL at C1D0, C1D6, and C2D0, respectively.

In the O3 group, bacterial load declined markedly between C1D0 and C1D6 (estimate = − 4.24; *P* < 0.001), with the median decreasing from 65,000 to 0 CFU/mL. The median relative reduction was 100% (IQR: −100.0 to − 99.9%), and the mean relative reduction in the original data scale was 99.6%. At C2D0, partial recrudescence was observed, with a median of 2,140 CFU/mL (IQR: 180–14,595), corresponding to a median relative reduction of 99.1% from baseline (IQR: −99.7 to − 62.8%). Although the increase from C1D6 to C2D0 was statistically significant (estimate = + 2.68; *P* < 0.001), values at C2D0 remained significantly below baseline (estimate = − 1.56; *P* < 0.001), with a mean bacterial load 77.5% lower than at C1D0 in the original data scale.

**Fig. 2:**
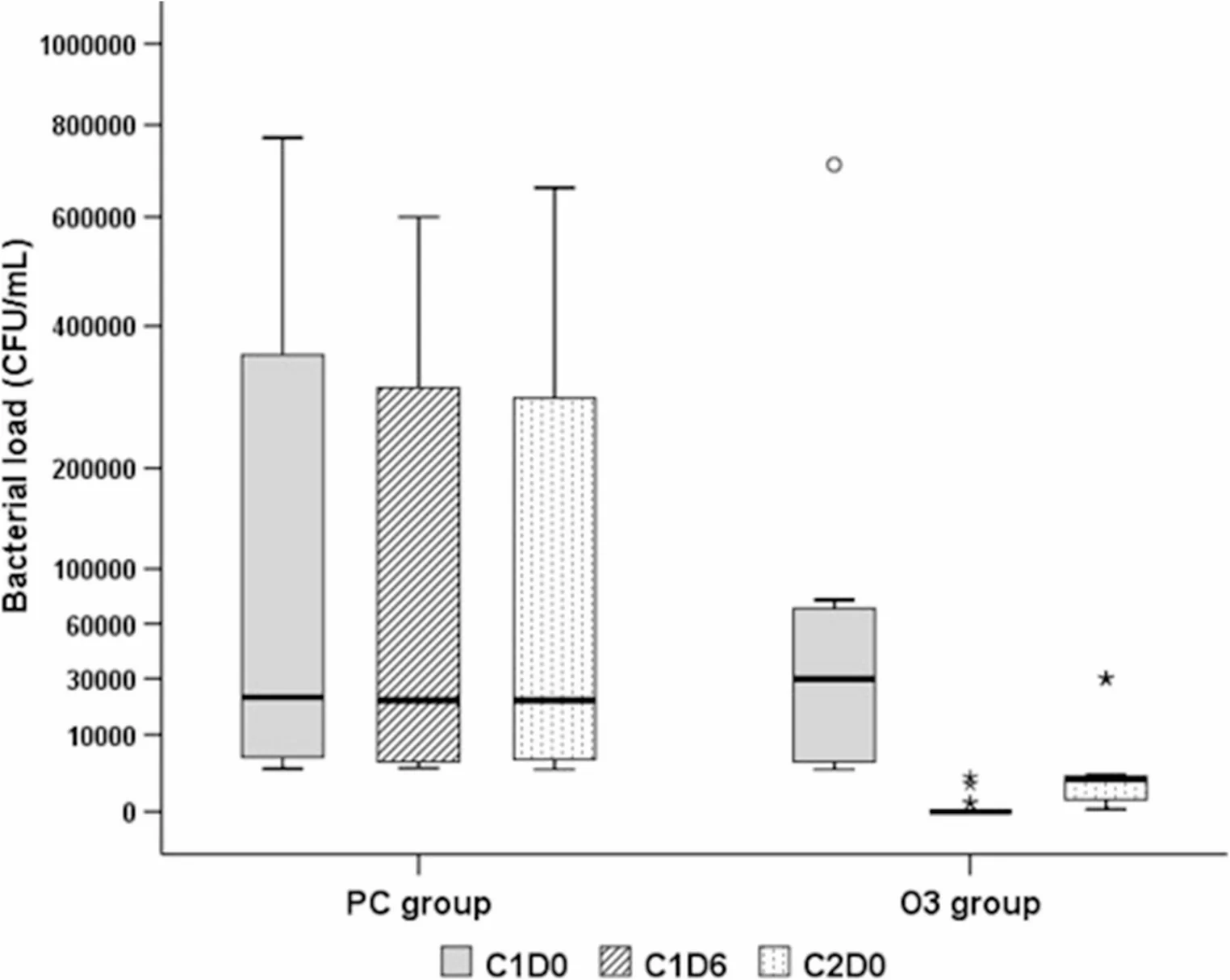
Longitudinal bacterial load in mares with mixed bacterial endometritis treated with intrauterine ozone therapy or pure medical oxygen. Boxplots represent bacterial load (CFU/mL) in the pure medical oxygen (PC; *n* = 6) and ozone therapy (O3; *n* = 9) groups at baseline (C1D0), six days post-treatment (C1D6), and at the onset of the subsequent estrous cycle (C2D0). The central line represents the median; the box represents the interquartile range (Q1–Q3); whiskers represent minimum and maximum values; and circles represent outliers. Significant differences were determined by predefined contrasts within the linear mixed-effects model (*P* < 0.05)

Between-group comparisons revealed significantly lower bacterial load in the O3 group relative to PC at C1D6 (estimate = 4.03; *P* < 0.001) and C2D0 (estimate = 1.35; *P* = 0.012) (Fig. 2).

Baseline neutrophil counts were comparable between groups (PC: median 10 cells/field, IQR 8.0–10.3; O3: median 10 cells/field, IQR 8.5–11.0; *P* = 0.87). The linear mixed model identified a significant group-by-time interaction, with significant interaction components at C1D6 (estimate = − 0.73; *P* < 0.001) and C2D0 (estimate = − 0.36; *P* < 0.001), indicating that neutrophil count trajectories differed significantly between groups across the experimental period.

In the PC group, neutrophil counts remained stable throughout follow-up, with no significant differences among time points (C1D0 vs. C1D6: *P* = 0.72; C1D0 vs. C2D0: *P* = 0.69; C1D6 vs. C2D0: *P* = 0.95) and medians of 10, 10, and 9.5 cells/field at C1D0, C1D6, and C2D0, respectively.

In the O3 group, neutrophil counts declined substantially between C1D0 and C1D6 (estimate = − 0.71; *P* < 0.001), with the median decreasing from 10 to 1 cell/field, corresponding to a relative reduction of 88.9% based on observed medians (IQR: −90.9 to − 67.5%). The mean relative reduction in the original data scale was 79.9%. At C2D0, values partially recovered to a median of 5 cells/field (IQR: 4.0–6.5), corresponding to a relative reduction of 50.0% from baseline (IQR: −61.8 to − 33.8%), remaining significantly below baseline values. The mean relative reduction at C2D0 was 42.4% relative to C1D0. The increase from C1D6 to C2D0 was statistically significant (estimate = + 0.37; *P* < 0.001), whereas values at C2D0 remained significantly below baseline (estimate = − 0.34; *P* < 0.001). Between-group comparisons revealed significantly lower neutrophil counts in the O3 group relative to PC at C1D6 (estimate = 0.75; *P* < 0.001) and C2D0 (estimate = 0.38; *P* < 0.001).

**Fig. 3:**
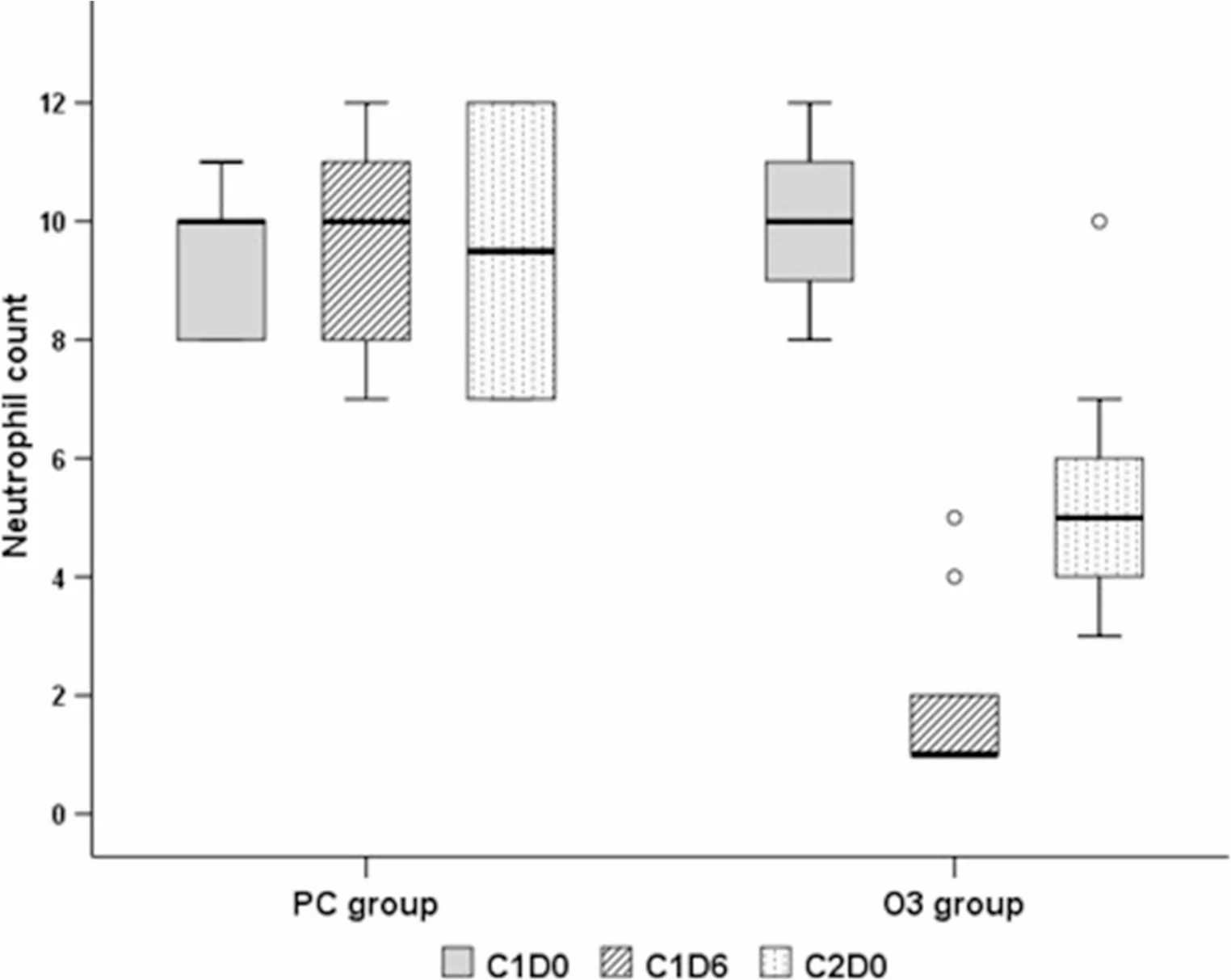
Longitudinal neutrophil counts in mares with mixed bacterial endometritis treated with intrauterine ozone therapy or pure medical oxygen. Boxplots represent neutrophil counts (cells/field) in the pure medical oxygen (PC; *n* = 6) and ozone therapy (O3; *n* = 9) groups at baseline (C1D0), six days post-treatment (C1D6), and at the onset of the subsequent estrous cycle (C2D0). The central line represents the median; the box represents the interquartile range (Q1–Q3); whiskers represent 1.5 times the interquartile range; and circles represent outliers. Significant differences were determined by predefined contrasts within the linear mixed-effects model (*P* < 0.05)

Complete descriptive statistics, including means, standard deviations, medians, interquartile ranges, and relative deltas stratified by group, are available in the supplementary material (Tables S1, S2A, and S2B) (Fig. 3).

## Discussion

The present study demonstrated that intrauterine ozone insufflation substantially reduced bacterial culture positivity, quantitative bacterial load, and endometrial inflammatory response in mares with naturally acquired mixed bacterial endometritis. Unlike most previous investigations, which relied primarily on qualitative microbiological outcomes, the present study combined qualitative bacterial identification with longitudinal quantification of uterine bacterial load (CFU/mL) across consecutive estrous cycles, supporting quantitative bacterial load as a valuable outcome measure for evaluating intrauterine therapies in mares.

The prevalence of mixed bacterial infections identified at screening was 29.8% (17/57), higher than reported by Davis et al. (2013; 12.6%) and Díaz-Bertrana et al. (2021; approximately 7% under field conditions). Part of this variation may reflect the sampling technique employed: unlike the double-guarded uterine swabs used in most prior work, the present study used low-volume uterine lavage, which samples a larger endometrial surface area and has been associated with greater diagnostic sensitivity (Ball et al. 1988; LeBlanc et al. 2007; Cocchia et al. 2012), consistent with the low mixed-infection rates reported in swab-based studies such as Ávila et al. (2022)d hne et al. (2023). These findings address a relevant methodological and clinical gap regarding the response of naturally occurring mixed bacterial infections to intrauterine ozone therapy.

The therapeutic complexity of mixed infections stems partly from the heterogeneous antimicrobial susceptibility of co-infecting organisms: Díaz-Bertrana et al. (2021) showed that Gram-positive and Gram-negative equine uterine isolates exhibit markedly distinct resistance patterns, such that a single antimicrobial protocol may fail to provide adequate coverage against all agents present, increasing the likelihood of incomplete clearance and recurrence. Ozone’s non-specific oxidative mechanism targets conserved structural components of both Gram-positive and Gram-negative bacteria regardless of individual susceptibility profile (Bocci et al. 2009; Donato et al. 2024). Accordingly, the pathological control group received pure medical oxygen rather than a standard-of-care comparator consisting of uterine lavage combined with antibiotic and/or ecbolic therapy. This design was intended to isolate the biological effects attributable to ozone from the confounding influence of concomitant antimicrobial treatment, providing a foundation for future comparative trials against conventional therapy. As such, the present findings should be interpreted as evidence of the biological efficacy of ozone itself, rather than as evidence of superiority over conventional therapeutic protocols.

Both groups presented comparable baseline microbiological and inflammatory characteristics, confirming group homogeneity before treatment. *E. coli*,* K. pneumoniae*,* Staphylococcus* spp., and *S. equi* subsp. *zooepidemicus* were the predominant isolates, consistent with pathogens classically associated with equine endometritis (LeBlanc and Causey 2009; Ferrer and Palomares 2018; Díaz-Bertrana et al. 2021). Bacterial load in the O3 group declined to a median of 0 CFU/mL at C1D6, accompanied by a marked reduction in neutrophil counts, while the PC group remained stable throughout, supporting the close association between bacterial control and resolution of the inflammatory response. Comparable microbiological improvement was reported by Ávila et al. (2022) following two days of O2-O3 insufflation, whereas Köhne et al. (2023), using a smaller gas volume at higher concentration, reported a more modest response, suggesting that total gas volume and duration of endometrial contact may influence therapeutic outcome more than concentration alone, given ozone’s short half-life (Bocci et al. 2009; Donato et al. 2024).

A methodologically relevant observation is that reliance on dichotomous culture positivity alone may underestimate the biological magnitude of the therapeutic response: although bacterial cultures became positive again in all mares of the O3 group at C2D0, quantitative bacterial load remained, on average, 77.5% below baseline values, a dissociation highlighting the limited resolution of binary classification, since a mare with a positive culture and low bacterial load represents a biologically distinct uterine environment from one with high microbial burden (LeBlanc et al. 2007; LeBlanc 2010). This supports longitudinal bacterial load quantification as a complementary endpoint in evaluating intrauterine therapies in mares.

At C1D6, the frequency of positive cultures in the O3 group did not differ from healthy controls (*P* = 0.260), whereas the PC group remained significantly different from Control (*P* = 0.0003), indicating that ozone temporarily restored a microbiological profile comparable to disease-free mares without inducing opportunistic colonization in healthy animals under the same protocol, consistent with the absence of adverse histological effects reported by Donato et al. (2023), Botelho et al. (2024), and Ferreira et al. (2021) following intrauterine ozone in healthy mares. Neutrophil counts followed a parallel pattern, consistent with bacterial persistence as the primary stimulus for neutrophil recruitment (LeBlanc and Causey 2009; Katila 2012). Likewise, the lack of microbiological and cytological improvement in the PC group suggests that uterine lavage and manipulation alone exerted, at most, a partial mechanical effect without relevant therapeutic impact, as also noted by Köhne et al. (2023). These findings support a direct therapeutic effect of ozone, potentially involving activation of inflammatory-resolution pathways such as the CD163 + macrophage response reported by Moroni et al. (2026) following intrauterine O2-O3 insufflation in subfertile mares.

The recolonization observed at C2D0, despite bacterial load and neutrophil counts remaining significantly below baseline, likely reflects incomplete rather than complete microbial eradication. One plausible explanation is that ozone substantially reduced, but did not completely eliminate, the bacterial population, allowing microorganisms persisting in deep endometrial niches, intracellular reservoirs, or biofilm-associated communities to recolonize the uterine lumen during the subsequent cycle (Petersen et al. 2015; Skive et al. 2017; Ferris 2022), a hypothesis reinforced by the qualitative pattern at C2D0, in which E. coli and K. pneumoniae remained the predominant isolates, closely resembling baseline.

Bacterial persistence, however, is unlikely to be the sole mechanism. Sample collection at C2D0 was intentionally performed at the onset of a new estrus, a stage characterized by cervical relaxation and increased uterine-vaginal communication that may favor recolonization even under appropriate reproductive management (Katila 2012); likewise, continuous environmental exposure and the manipulations inherent to the commercial embryo transfer program in which mares remained enrolled cannot be excluded as additional contributors. These findings are also compatible with a predominantly suppressive rather than fully eradicative effect of ozone: given its immediate oxidative action and lack of residual antimicrobial activity once the gas dissipates (Bocci et al. 2009; Donato et al. 2024), microorganisms shielded from exposure during treatment may subsequently recolonize the lumen once the oxidative stimulus ceases.

Because molecular strain characterization was not performed, persistence of the original infection could not be distinguished from reinfection by distinct strains (Rasmussen et al. 2013). The recurrence at C2D0 therefore likely reflects a multifactorial process combining bacterial persistence, physiological recolonization, and possible environmental re-exposure; future studies incorporating molecular strain typing, extended follow-up, and multi-cycle protocols will be important to clarify these mechanisms.

From a clinical perspective, the period immediately following treatment, marked by pronounced reductions in bacterial load and neutrophil infiltration, may represent a favorable therapeutic window for procedures such as artificial insemination or embryo transfer, a hypothesis warranting direct evaluation in prospective fertility studies (Ávila et al. 2022; Köhne et al. 2023).

Several limitations should be acknowledged. Sample size, constrained by the lower prevalence of naturally acquired mixed bacterial infections, limited statistical power for subgroup analyses by bacterial species. Because bacterial endometritis is ultimately a fertility-limiting condition, the absence of fertility outcomes (pregnancy rate, embryo recovery, or conception rate) represents an important limitation: the present findings demonstrate microbiological and anti-inflammatory efficacy but cannot establish whether these improvements translate into enhanced reproductive performance. Direct comparison with standard-of-care therapy was likewise not performed and remains an important next step in establishing the comparative clinical value of ozone relative to conventional treatment.

The healthy control group had a lower mean age than the endometritis groups but was included exclusively for microbiological and inflammatory safety monitoring in a disease-free endometrium, with all primary treatment-response analyses conducted exclusively between the PC and O3 groups. Inflammatory status was classified by cytology and microbiology alone, without histopathological evaluation; culture-based methods likewise do not allow complete characterization of the uterine microbiota or detection of anaerobic, biofilm-associated, or non-cultivable microorganisms potentially involved in chronic endometritis (Canisso et al. 2020; Ferris 2022). Finally, the study population, subfertile Mangalarga Marchador embryo recipient mares, may limit generalizability to other breeds and clinical contexts.

Despite these limitations, this is the first study to specifically evaluate intrauterine ozone therapy in mares with naturally acquired mixed bacterial endometritis using longitudinal CFU/mL quantification as the primary outcome. The present findings demonstrate biological activity of intrauterine ozone therapy against naturally acquired mixed bacterial endometritis while supporting longitudinal bacterial load quantification as a valuable outcome measure for evaluating intrauterine therapies in mares. Future studies with larger sample sizes, multi-cycle protocols, molecular strain typing, histopathological evaluation, and reproductive outcome monitoring, including direct comparison with conventional therapy, are warranted to consolidate these findings before ozone therapy can be recommended as a routine therapeutic strategy for mares with mixed bacterial endometritis.

In conclusion, intrauterine ozone insufflation at 42 µg/mL reduced bacterial load, microbiological culture positivity, and endometrial neutrophil infiltration in mares with naturally acquired mixed bacterial endometritis. Although bacterial recrudescence occurred in the subsequent estrous cycle, bacterial load and neutrophil counts remained significantly below baseline values, indicating a partial but sustained therapeutic effect following a single treatment cycle.

The absence of bacterial growth in clinically healthy mares subjected to the same protocol supports the microbiological safety of intrauterine ozone therapy under the conditions evaluated. Taken together, these findings indicate that intrauterine ozone insufflation warrants further investigation as a therapeutic strategy for mixed bacterial infections in mares, particularly regarding multi-cycle protocols and reproductive outcome assessment.

## Supplementary Information

Below is the link to the electronic supplementary material.

Supplementary Material 1

## Data Availability

The datasets generated and/or analyzed during the current study are available from the corresponding author on reasonable request.
