# Supplementary material for: Intrauterine ozone therapy reduces bacterial load in mares with mixed bacterial endometritis: a longitudinal study
Source: Vet Res Commun. 2026 Jul 31;50(5):497. doi: 10.1007/s11259-026-11438-3 (PMC13427915; doi:10.1007/s11259-026-11438-3)
Supplement: Supplementary file 1 — Supplementary Material 1 [file 11259_2026_11438_MOESM1_ESM.docx]

**Table S1.** Uterine bacterial culture positivity according to experimental group at baseline (C1D0), six days after treatment (C1D6), and at the onset of the subsequent estrous cycle (C2D0).

| **Variable** | **Control Group** | | **PC Group** | | **O3 Group** | | **p value**  Control × PC | **p value**  Control × O3 | **p value**  PC × O3 |
| --- | --- | --- | --- | --- | --- | --- | --- | --- | --- |
|  | **n** | **%** | **n** | **%** | **n** | **%** |  |  |  |
| Bacterial culture positivity - C1D0 | 0 | 0 | 6 | 100 | 9 | 100 | 0.0003 | < 0.0001 | 1 |
| Bacterial culture positivity - C1D6 | 0 | 0 | 6 | 100 | 2 | 22.2 | 0.0003 | 0.26 | 0.005 |
| Bacterial culture positivity - C2D0 | 0 | 0 | 6 | 100 | 9 | 100 | 0.0003 | < 0.0001 | 1 |
|  | | | | | | | | | |

Bacterial culture positivity expressed as absolute (n) and relative (%) frequency. Comparisons between groups at each time point were performed using Fisher's exact test. In the O3 group, McNemar's exact test indicated significant temporal variation between time points C1D0 and C1D6 (p = 0.015) and between C1D6 and C2D0 (p = 0.015). The PC group showed no variation over time. Control: healthy mares submitted to intrauterine ozone insufflation (n = 8); PC: mares with mixed bacterial infection treated with pure medicinal oxygen (n = 6); O3: mares with mixed bacterial infection treated with intrauterine ozone therapy (n = 9).

**Table S2A.** Descriptive statistics of bacterial load (CFU/mL) and neutrophil count variables in the PC group (pure medicinal oxygen; n = 6) at baseline (C1D0), six days after treatment (C1D6), and at the onset of the subsequent estrous cycle (C2D0), with respective relative deltas between time points.

|  |  |  |  |  |  |  |  |  |  |
| --- | --- | --- | --- | --- | --- | --- | --- | --- | --- |
|  |  |  |  |  |  |  |  |  |  |
| **Variable** | **n** | **mean** | **SD** | **median** | **IQR** | | | **minimum** | **maximum** |
| Idade (anos) | 6 | 11,5 | 1,9 | 11,5 | 9,75 | - | 13,3 | 9 | 14 |
|  |  |  |  |  |  |  |  |  |  |
| CFU/mL - C1D0 | 6 | 124467 | 215298 | 51550 | 6475 | - | 193250 | 6100 | 560000 |
| CFU/mL - C1D6 | 6 | 131367 | 226688 | 54750 | 6925 | - | 203750 | 6700 | 590000 |
| CFU/mL - C2D0 | 6 | 140133 | 246869 | 51250 | 6975 | - | 223750 | 6000 | 640000 |
|  |  |  |  |  |  |  |  |  |  |
| Δ% (C1D6 -C1D0) | 6 | 7,0 | 4,7 | 5,5 | 3,6 | - | 11,4 | 1,5 | 14,8 |
| Δ% (C2D0- C1D0) | 6 | 8,5 | 13,1 | 13,8 | -7,6 | - | 19,7 | -9,1 | 19,7 |
|  |  |  |  |  |  |  |  |  |  |
| Neutrophils - C1D0 | 6 | 9,5 | 1,2 | 10 | 8 | - | 10,3 | 8 | 11 |
| Neutrophils- C1D6 | 6 | 9,7 | 2,0 | 10 | 7,8 | - | 11,3 | 7 | 12 |
| Neutrophils - C2D0 | 6 | 9,5 | 2,3 | 9,5 | 7 | - | 12 | 7 | 12 |
|  |  |  |  |  |  |  |  |  |  |
| Δ% (C1D6 -C1D0) | 6 | 3,2 | 24,8 | 5,0 | -21,1 | - | 24,4 | -30,0 | 37,5 |
| Δ% (C2D0- C1D0) | 6 | 1,6 | 29,2 | -6,3 | -21,1 | - | 27,5 | -30,0 | 50,0 |

SD: standard deviation; IQR: interquartile range (Q1–Q3). Δ%: relative percentage change from baseline (C1D0).

**Table S2B.** Descriptive statistics of bacterial load (CFU/mL) and neutrophil count variables in the O3 group (intrauterine ozone therapy; n = 9) at baseline (C1D0), six days after treatment (C1D6), and at the onset of the subsequent estrous cycle (C2D0), with respective relative deltas between time points.

|  |  |  |  |  |  |  |  |  |  |
| --- | --- | --- | --- | --- | --- | --- | --- | --- | --- |
|  |  |  |  |  |  |  |  |  |  |
| **Variable** | **n** | **mean** | **SD** | **median** | **IQR** | | | **minimum** | **maximum** |
| Age (years) | 9 | 10,6 | 2,5 | 10 | 8,5 | - | 13 | 7 | 14 |
|  |  |  |  |  |  |  |  |  |  |
| CFU/mL - C1D0 | 9 | 151367 | 237550 | 65000 | 33050 | - | 191000 | 6100 | 740000 |
| CFU/mL - C1D6 | 9 | 126 | 331 | 0 | 0 | - | 65 | 0 | 1000 |
| CFU/mL - C2D0 | 9 | 43922 | 119217 | 2140 | 180 | - | 14595 | 150 | 361000 |
|  |  |  |  |  |  |  |  |  |  |
| Δ% (C1D6 -C1D0) | 9 | -99,6 | 1,0 | -100,0 | -100,0 | - | -99,9 | -100,0 | -97,0 |
| Δ% (C2D0- C1D0) | 9 | -77,5 | 39,6 | -99,1 | -99,7 | - | -62,8 | -99,8 | 20 |
|  |  |  |  |  |  |  |  |  |  |
| Neutrophils - C1D0 | 9 | 9,8 | 1,4 | 10 | 8,5 | - | 11 | 8 | 12 |
| Neutrophils- C1D6 | 9 | 1,9 | 1,5 | 1 | 1 | - | 3 | 1 | 5 |
| Neutrophils - C2D0 | 9 | 5,4 | 2,1 | 5 | 4 | - | 6,5 | 3 | 10 |
|  |  |  |  |  |  |  |  |  |  |
| Δ% (C1D6 -C1D0) | 9 | -79,9 | 17,0 | -88,9 | -90,9 | - | -67,5 | -91,7 | -44,4 |
| Δ% (C2D0- C1D0) | 9 | -42,4 | 28,0 | -50,0 | -61,8 | - | -33,8 | -66,7 | 25,0 |

SD: standard deviation; IQR: interquartile range (Q1–Q3). Δ%: relative percentage change from baseline (C1D0).
